# Association between previous negative biopsies and lower rates of progression during active surveillance for prostate cancer

**DOI:** 10.1007/s00345-022-03983-8

**Published:** 2022-03-26

**Authors:** Mattia Luca Piccinelli, Stefano Luzzago, Giulia Marvaso, Ekaterina Laukhtina, Noriyoshi Miura, Victor M. Schuettfort, Keiichiro Mori, Alberto Colombo, Matteo Ferro, Francesco A. Mistretta, Nicola Fusco, Giuseppe Petralia, Barbara A. Jereczek-Fossa, Shahrokh F. Shariat, Pierre I. Karakiewicz, Ottavio de Cobelli, Gennaro Musi

**Affiliations:** 1 Department of Urology, IEO European Institute of Oncology, IRCCS, Via Giuseppe Ripamonti 435, Milan, Italy; 2 Università degli Studi di Milano, Milan, Italy; 3 Department of Radiotherapy, IEO European Institute of Oncology, IRCCS, Via Ripamonti 435, Milan, Italy; 4 Institute for Urology and Reproductive Health, Sechenov University, Moscow, Russia; 5 Department of Urology, Medical University of Vienna, Vienna, Austria; 6 Department of Urology, Ehime University Graduate School of Medicine, Ehime, Japan; 7 Department of Urology, University Medical Center Hamburg Eppendorf, Hamburg, Germany; 8 Department of Urology, The Jikei University School of Medicine, Tokyo, Japan; 9 Precision Imaging and Research Unit, Department of Medical Imaging and Radiation Sciences, IEO European Institute of Oncology IRCCS, 20141 Milan, Italy; 10 Division of Radiology, IEO European Institute of Oncology, IRCCS, Via Ripamonti 435, Milan, Italy; 11 Department of Pathology, IEO European Institute of Oncology, IRCCS, Via Ripamonti 435, Milan, Italy; 12 Department of Oncology and Haemato-Oncology, Università Degli Studi Di Milano, 20122 Milan, Italy; 13 Research Division of Urology, Department of Special Surgery, The University of Jordan, Amman, Jordan; 14 Department of Urology, University of Texas Southwestern Medical Center, Dallas, TX USA; 15 Department of Urology, Second Faculty of Medicine, Charles University, Prague, Czech Republic; 16 Department of Urology, Weill Cornell Medical College, New York, NY USA; 17 Karl Landsteiner Institute of Urology and Andrology, Vienna, Austria; 18 European Association of Urology Research Foundation, Arnhem, Netherlands; 19 Cancer Prognostics and Health Outcomes Unit, Division of Urology, University of Montréal Health Center, Montréal, QC Canada

**Keywords:** Active surveillance, Previous negative biopsies, Biopsy naïve, Any-cause discontinuation, Upgrading

## Abstract

**Purpose:**

To test any-cause discontinuation and ISUP GG upgrading rates during Active Surveillance (AS) in patients that underwent previous negative biopsies (PNBs) before prostate cancer (PCa) diagnosis vs. biopsy naive patients.

**Methods:**

Retrospective analysis of 961 AS patients (2008–2020). Three definitions of PNBs were used: (1) PNBs status (biopsy naïve vs. PNBs); (2) number of PNBs (0 vs. 1 vs. ≥ 2); (3) histology at last PNB (no vs. negative vs. HGPIN/ASAP). Kaplan–Meier plots and multivariable Cox models tested any-cause and ISUP GG upgrading discontinuation rates.

**Results:**

Overall, 760 (79.1%) vs. 201 (20.9%) patients were biopsy naïve vs. PNBs. Specifically, 760 (79.1%) vs. 138 (14.4%) vs. 63 (6.5%) patients had 0 vs. 1 vs. ≥ 2 PNBs. Last, 760 (79.1%) vs. 134 (13.9%) vs. 67 (7%) patients had no vs. negative PNB vs. HGPIN/ASAP. PNBs were not associated with any-cause discontinuation rates. Conversely, PNBs were associated with lower rates of ISUP GG upgrading: (1) PNBs vs. biopsy naïve (HR:0.6, *p* = 0.04); (2) 1 vs. 0 PNBs (HR:0.6, *p* = 0.1) and 2 vs. 0 PNBs, (HR:0.5, *p* = 0.1); (3) negative PNB vs. biopsy naïve (HR:0.7, *p* = 0.3) and HGPIN/ASAP vs. biopsy naïve (HR:0.4, *p* = 0.04). However, last PNB ≤ 18 months (HR:0.4, *p* = 0.02), but not last PNB > 18 months (HR:0.8, *p* = 0.5) were associated with lower rates of ISUP GG upgrading.

**Conclusion:**

PNBs status is associated with lower rates of ISUP GG upgrading during AS for PCa. The number of PNBs and time from last PNB to PCa diagnosis (≤ 18 months) appear also to be critical for patient selection.

**Supplementary Information:**

The online version contains supplementary material available at 10.1007/s00345-022-03983-8.

## Introduction

Active surveillance (AS) represents a valid management strategy for patients with very low- and low-volume intermediate-risk prostate cancer (PCa) [1–5]. However, previous series showed a risk of disease misclassification, at the time of AS beginning that ranges from 22 to 33% [2]. Moreover, even the most stringent AS criteria are not able to fully discriminate patients affected by clinically significant PCa (csPCa) [6]. Novel tools have been tested to reduce disease misclassification rates. For example, confirmatory biopsies or multiparametric magnetic resonance imaging (mpMRI) have been employed in daily practice [7–14].

Previous negative biopsies (PNBs), before PCa diagnosis, have been associated with lower rates of adverse findings at radical prostatectomy (RP) [15–19].

We hypothesized lower rates of disease progression during AS in PNBs patients, relative to their biopsy naïve counterparts.

To address this void, we focussed on a large contemporary series of AS patients and we tested two commonly used AS outcomes, namely: (1) any-cause discontinuation; (2) discontinuation due to upgrading.

## Materials and methods

### Study population

This retrospective single-institution data analysis was approved by the Institutional Review Board of the European Institute of Oncology.

Overall, 961 patients with PCa were enrolled in AS between 2008 and 2020. AS inclusion criteria were the following: prostate specific antigen (PSA) ≤ 10 ng/ml; clinical stage (cT) cT1c/cT2a; International Society of Urological Pathology Grade Group (ISUP GG) 1 PCa with ≤ 3 positive cores or ISUP GG2 PCa with pattern 4 < 10% in a single core; PSA-density (PSAD) < 0.2 ng/ml/ml. AS protocol consisted of: repeated PSA testing (every 6 months for 5 years and annually thereafter); clinical assessment every 12 months and repeated surveillance biopsies scheduled at 12, 36 and 84 months. Since 2015, confirmatory mpMRI was employed in AS protocol and offered to all patients at AS begin (≤ 6 months from diagnostic biopsy). Here, all patients with Prostate Imaging Reporting and Data System (PI-RADS) score ≥ 3 underwent confirmatory targeted biopsies, as previously reported [20, 21]. Additionally, repeated mpMRI scans were performed before surveillance biopsies. Here, 1–3 targeted-cores were additionally taken in patients with positive mpMRIs (PI-RADS score ≥ 3) [22–24].

Patients were switched to active treatment (AT) due to: (1) ISUP GG upgrading (ISUP GG ≥ 2 with > 10% of pattern 4); (2) volume upstaging (> 3 positive cores with ISUP GG1 PCa); (3) rising PSA; (4) suspicious extra-prostatic extension at mpMRI; (5) patient preference.

### Variables of interest

We tested the association between PNBs and AS outcomes. Specifically, PNBs were defined as all negative prostate samplings performed before the diagnostic biopsy that allowed for AS begin. Three different definitions of PNBs were used: (1) PNBs status (biopsy naïve vs. PNBs); (2) number of PNBs (0 [biopsy naïve] vs. 1 vs. ≥ 2); (3) histology at last PNB before the diagnosis of PCa that allowed for AS begin (biopsy naïve vs. negative vs. high-grade prostatic intraepithelial neoplasia [HGPIN] and/or atypical small acinar proliferation [ASAP]).

### Statistical analyses

We focussed on two AS outcomes: (1) any-cause discontinuation; (2) discontinuation due to ISUP GG upgrading (ISUP GG ≥ 2 with > 10% of pattern 4).

Differences in medians and proportions were evaluated by, respectively, the Kruskal–Wallis and chi-square tests. First, Kaplan–Meier (KM) plots tested any-cause discontinuation and ISUP GG upgrading rates over time. Second, multivariable Cox regression models tested associations between patient’s or tumour characteristics and rates of AS discontinuation.

R software environment was used in all statistical analyses and graphics (version 3.4.3). All tests were two sided with a level of significance set at *p* < 0.05.

## Results

### Descriptive analyses (Table 1)

**Table 1:** Clinical characteristics and findings at diagnostic biopsy of 961 patients enrolled in AS between 2008 and 2020

	Overall (*n* = 961)	Biopsy naïve (*n* = 760; 79.1)	PNBs (*n* = 201; 20.9)	*p* value
Age (years) median (IQR)	64 (59–69)	64 (58–69)	65 (61–69)	**0.04**
PSA (ng/ml) median (IQR)	5.8 (4.4–7.6)	5.6 (4.3–7.4)	6.5 (5.1–9.2)	** < 0.001**
PSAD (ng/ml/ml) median (IQR)	0.1 (0.1–0.2)	0.1 (0.1–0.2)	0.1 (0.1–0.2)	0.6
cT				0.7
cT1c	880 (91.6)	698 (91.8)	182 (90.5)	
cT2a	81 (8.4)	62 (8.2)	19 (9.5)	
Confirmatory mpMRI				0.09
No	268 (27.9)	202 (26.6)	66 (32.8)	
Yes	693 (72.1)	558 (73.4)	135 (67.2)	
Diagnostic biopsy cores median (IQR)	13 (12–16)	13 (12–16)	14 (12–18)	0.2
Diagnostic biopsy positive cores				** < 0.001**
1	594 (61.8)	444 (58.4)	150 (74.6)	
2	254 (26.4)	212 (27.9)	42 (20.9)	
3	113 (11.8)	104 (13.7)	9 (4.5)	
ISUP GG				1
1	930 (96.8)	736 (96.8)	194 (96.5)	
2	31 (3.2)	24 (3.2)	7 (3.5)	
Number of PNBs				** < 0.001**
0 (biopsy naïve)	760 (79.1)	760 (100)	0 (0)	
1	138 (14.4)	0 (0)	138 (68.7)	
≥ 2	63 (6.5)	0 (0)	63 (31.3)	
Histology at last PNB				** < 0.001**
Biopsy naïve	760 (79.1)	760 (79.1)	0 (0)	
Negative	134 (13.9)	0 (0)	134 (13.9)	
HGPIN/ASAP	67 (7)	0 (0)	67 (7)	

Stratification is made according to PNBs status (biopsy naïve vs. PNBs). Data are shown as medians for continuous variables or as counts and percentages (%) for categorical variables

*PNBs* previous negative biopsies, *AS* active surveillance, *IQR* interquartile range, *PSA* prostate specific antigen, *PSAD* prostate specific antigen density, *cT* clinical T stage, *mpMRI* multiparametric magnetic resonance imaging, *ISUP* *GG* international society of urological pathology grade group, *HGPIN* high-grade prostatic intraepithelial neoplasia, *ASAP* atypical small acinar proliferation

Bold values indicate statistical significance *p* < 0.05

Overall, 760 (79.1%) vs. 201 (20.9%) patients were biopsy naïve vs. PNBs. Specifically, 760 (79.1%) vs. 138 (14.4%) vs. 63 (6.5%) patients had 0 (biopsy naïve) vs. 1 vs. ≥ 2 PNBs. Last, according to histology at last PNB, 760 (79.1%) vs. 134 (13.9%) vs. 67 (7%) patients had no PNBs (biopsy naïve) vs. negative PNB vs. HGPIN/ASAP.

PNBs patients were older (median: 65 vs. 64 years, *p* = 0.04) and had higher median PSA at AS begin (6.5 vs. 5.6 ng/ml, *p* < 0.001), relative to their biopsy naïve counterparts. Moreover, PNBs patients had less frequently two (20.9 vs. 27.9%) or three (4.5 vs. 13.7%) positive cores at diagnostic biopsy (*p* < 0.001).

### Findings at follow-up

Median (IQR: interquartile range) time follow-up was 35 (18–63) months. During AS, 32.8 vs. 44.1%, 16.4 vs. 15% and 10 vs. 6.8% PNBs vs. biopsy naïve patients underwent 1, 2, ≥ 3 surveillance biopsies, respectively (*p* = 0.03).

Overall, 34.3 vs. 34.5% (*p* = 0.9) PNBs vs. biopsy naïve patients experienced any-cause discontinuation. Conversely, 10.9 vs. 15.8% (*p* = 0.1) PNBs vs. biopsy naïve patients had ISUP GG upgrading (Supplementary Table 1).

**Fig. 1:**
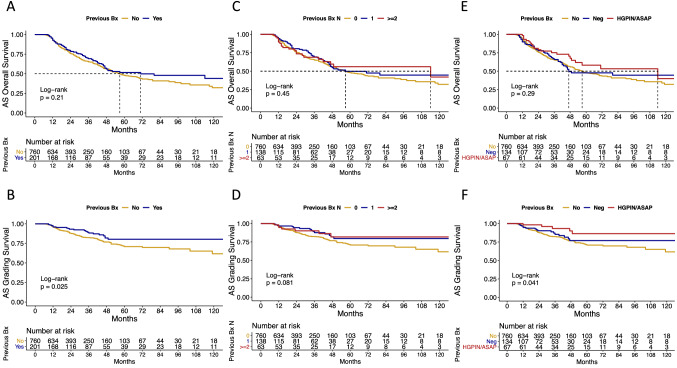
Kaplan–Meier plots with log-rank test depicting any-cause and ISUP GG upgrading survival over time, according to PNBs definition: **a** any-cause survival in biopsy naïve vs. PNBs patients; **b** ISUP GG upgrading survival in biopsy naïve vs. PNBs patients; **c** any-cause survival in patients with 0 (biopsy naïve) vs. 1 vs. ≥ 2 PNBs; **d** ISUP GG upgrading survival in patients with 0 (biopsy naïve) vs. 1 vs. ≥ 2 PNBs; **e** any-cause survival in biopsy naïve patients vs. patients with negative PNBs vs. patients with HGPIN/ASAP; **f** ISUP GG upgrading survival in biopsy naïve patients vs. patients with negative PNBs vs. patients with HGPIN/ASAP *AS* active surveillance, *PNBs* previous negative biopsies, *ISUP GG* international society of urological pathology grade group, *HGPIN* high-grade prostatic intraepithelial neoplasia, *ASAP* atypical small acinar proliferation

In KM plots, 3-year rates of any-cause and ISUP GG upgrading survival were, respectively, 70 vs. 65% (*p* = 0.2; Fig. 1a) and 88 vs. 82% (*p* = 0.02; Fig. 1b) in PNBs vs. biopsy naïve patients. Moreover, 3-year rates of any-cause and ISUP GG upgrading survival were, respectively, 65% vs. 69% vs. 69% (*p* = 0.4; Fig. 1c) and 82% vs. 87% vs. 90% (*p* = 0.08; Fig. 1d) in patients with 0 vs. 1 vs. ≥ 2 PNBs. Last, 3-year rates of any-cause and ISUP GG upgrading survival were, respectively, 65% vs. 66% vs. 75% (*p* = 0.2; Fig. 1e) and 82% vs. 85% vs. 93% (*p* = 0.04; Fig. 1f) in biopsy naïve patients vs. patients with negative PNBs vs. patients with HGPIN/ASAP.

In multivariable Cox regression models (Table 2), PNBs were not associated with lower rates of any-cause discontinuation, regardless of PNBs definition. Conversely, in multivariable Cox regression models, PNBs were associated with lower rates of ISUP GG upgrading: (1) PNBs vs. biopsy naïve, Hazard Ratio (HR):0.6 (95% confidence interval [CI]:0.4–0.9, *p* = 0.04); (2) 1 vs. 0 PNBs, HR:0.6 (95% CI:0.4–1.1, *p* = 0.1) and 2 vs. 0 PNBs, HR:0.5 (95% CI:0.3–1.1, *p* = 0.1); (3) negative PNB vs. biopsy naïve, HR:0.7 (95% CI:0.4–1.3, *p* = 0.3) and HGPIN/ASAP vs. biopsy naïve, HR:0.4 (95% CI:0.2–0.9, *p* = 0.04).

**Table 2:** Separate multivariable Cox-regression models predicting (a) any-cause AS discontinuation rates and (b) ISUP GG upgrading rates, according to PNBs status (yes vs. no), number of PNBs at AS begin (0 [biopsy naïve] vs. 1 vs. ≥ 2) and histology at last PNB before AS diagnostic biopsy (biopsy naïve vs. negative vs. HGPIN/ASAP)

	Hazard ratio (HR) [95% CI]	*p* value
(a) Any-cause AS discontinuation rates		
PNBs (yes vs. no)	0.9 (0.6–1.2)	0.4
Number of PNBs		
0 (biopsy naïve)	Ref.	
1	0.9 (0.65–1.2)	0.5
≥ 2	0.9 (0.5–1.4)	0.5
Histology at last PNB		
Biopsy naïve	Ref.	
Negative	0.9 (0.7–1.3)	0.7
HGPIN/ASAP	0.8 (0.5–1.2)	0.3
(b) ISUP GG upgrading rates		
PNBs (yes vs. no)	0.6 (0.4–0.9)	**0.04**
Number of PNBs		
0 (biopsy naïve)	Ref.	
1	0.6 (0.4–1.1)	0.1
≥ 2	0.5 (0.3–1.1)	0.1
Histology at last PNB		
Biopsy naïve	Ref.	
Negative	0.7 (0.4–1.3)	0.3
HGPIN/ASAP	0.4 (0.2–0.9)	**0.04**

All models are adjusted for clinical characteristics and biopsy findings at patient enrolment: age (years), PSA (ng/ml), cT (cT1c vs. cT2a), confirmatory mpMRI (no vs. yes), number of positive cores at biopsy (1 vs. 2 vs. 3), ISUP GG at biopsy (1 vs. 2). Bold values indicate statistical significance* p* < 0.05

*AS* active surveillance, *PNBs* previous negative biopsies, *HGPIN* high-grade prostatic intraepithelial neoplasia, *ASAP* atypical small acinar proliferation, *PSA* prostate specific antigen, *cT* clinical T stage, *mpMRI* multiparametric magnetic resonance imaging, *ISUP GG* international society of urological pathology grade group, *CI* confidence interval

### Sensitivity analysis: time between last PNB and AS begin

We performed a sensitivity analysis to test the association between time from last PNB to AS begin and the two mentioned outcomes. Median (IQR) time from last PNB to AS begin was 19 (9–40) months. In KM plots, 3-year rates of any-cause and ISUP GG upgrading survival were, respectively, 65% vs. 65% vs. 74% (*p* = 0.1; Supplementary Fig. 1a) and 82% vs. 82% vs. 93% (*p* = 0.02; Supplementary Fig. 1b) in biopsy naïve patients vs. last PNB > 18 months vs. last PNB ≤ 18 months.

In multivariable Cox regression models (Supplementary Table 2), neither last PNB ≤ 18 months (HR:0.7 [95% CI:0.5–1.05, *p* = 0.09]) nor last PNB > 18 months (HR:1.1 [95% CI:0.7–1.5, *p* = 0.8]) were associated with lower rates of any-cause discontinuation. Conversely, last PNB ≤ 18 months (HR:0.4 [95% CI:0.2–0.9, *p* = 0.02]), but not last PNB > 18 months (HR:0.8 [95% CI:0.4–1.5, *p* = 0.5]) were associated with lower rates of ISUP GG upgrading, relative to biopsy naïve patients.

## Discussion

Patient selection at AS begin represents a critical step. To date, novel confirmatory exams have been developed and implemented in daily practice [7–14, 25–29]. However, most of these tools are usually performed several months after PCa diagnosis [7–11]. Moreover, possible side effects or elevated costs [25–27] limit patient compliance. In consequence, there is an urgent need to find immediately available and low-cost tools to confirm patient eligibility to AS. We focussed on the association between PNBs and two commonly used AS outcomes, namely any-cause and ISUP GG upgrading discontinuation, in a large series of AS patients. Specifically, the protective effect of PNBs was tested in a systematic fashion and accordingly to three PNBs definitions, namely: (1) PNBs status (biopsy naïve vs. PNBs); (2) number of PNBs (0 [biopsy naïve] vs. 1 vs. ≥ 2); (3) histology at last PNB (biopsy naïve vs. negative histology vs. HGPIN/ASAP). Our results showed several important findings.

First, we observed similar rates of any-cause discontinuation between biopsy naïve and PNBs patients, regardless of PNBs definition. However, when ISUP GG upgrading (ISUP GG ≥ 2 with > 10% of pattern 4) rates were tested, PNBs patients were at lower risk of upgrading (HR:0.6; *p* = 0.04), relative to their biopsy naïve counterparts. Moreover, while we were unable to reach statistical significance (*p* = 0.1) due to low number of patients, a gradual increase in the protective association between PNBs and ISUP GG upgrading rates was observed with an increasing number of PNBs (HR:0.6 for 1 PNB; HR:0.5 for ≥ 2 PNBs). Last, histology at last PNB was also associated with ISUP GG upgrading rates. Specifically, only patients with HGPIN/ASAP (HR:0.4; *p* = 0.04), but not patients with negative findings at last PNB (HR:0.7; *p* = 0.3), were at lower risk of ISUP GG upgrading over time. Our results are supported by the non-negligible follow-up time (35 months) and by the use of multivariable Cox regression models that were fully adjusted for all available patients and tumour characteristics. To the best of our knowledge, we are the first to specifically focus on the role of PNBs on AS outcomes. Indeed, several previous authors tested the association between findings at confirmatory biopsy (≤ 1 year after PCa diagnosis) and AS outcomes over time [7–11], without considering prostate samplings before AS begin. Due to the novelty of our analysis, only hypothetical considerations could justify our findings. Specifically, we can hypothesize that PNBs patients could be at lower risk of disease misclassification at AS enrolment. This hypothesis is supported by several previous manuscripts that reported lower rates of csPCa at RP in patients that underwent multiple prostate samplings before surgery [15–19]. Indeed, Rosenbaum et al. and Djavan et al. observed decreasing rates of extra-prostatic PCa, aggressive tumour histology and lymph node metastases with an increasing number of prostate biopsies before RP [16, 17]. Our results could be used for patients counselling at AS recruitment. Specifically, the use of AS confirmatory tests could be modulated according to PNBs status. Indeed, confirmatory biopsies could be recommended for all patients that were biopsy naïve before PCa diagnosis. Conversely, due to the retrospective nature of the current analysis, the modulation of AS follow-up schemes, in which PNBs patients could be less frequently submitted to repeated biopsies, should be tested in other multi-institutional and ideally prospective studies.

Second, when we repeated our analyses after considering time from last PNB to AS begin, we observed that only patients with last PNB performed ≤ 18 months (HR:0.4; *p* = 0.02), but not patients with last PNB > 18 months (HR:0.8; *p* = 0.5) were at lower risk of ISUP GG upgrading. We can argue that only patients with at least one PNB ≤ 18 months are at lower risk of disease misclassification at AS begin. Indeed, prostate samplings performed > 18 months could not be representative of patient situation at the moment of PCa diagnosis. Moreover, these findings could also justify the observed association between histology at last PNB and ISUP GG upgrading rates, where only patients with HGPIN/ASAP, but not patients with negative PNB, were at lower risk of ISUP GG upgrading. Indeed, during the study period, the EAU guidelines recommended prompt prostate re-biopsy for all patients diagnosed with HGPIN/ASAP [1]. In consequence, it is possible that the observed association between HGPIN/ASAP at last PNB and lower rates of ISUP GG upgrading over time is not related to biopsy histology but is a product of time from last PNB.

Taken together, PNBs history appears to be a useful tool for confirming patient eligibility to AS. Specifically, PNBs patients are at lower risk of disease upgrading during AS, relative to their biopsy naïve counterparts. This protective association appears to be even stronger in patients with a history of multiple PNBs before PCa diagnosis. However, time from last PNB to AS begin should be considered as a critical factor. Results from other series, with a specific focus on RP findings after AS, are warranted before recommending AS protocols modifications.

Despite its novelty our study has limitations. First, the current data are retrospective and influenced by inherent selection bias. Second, the long-time span (2008–2020) of the current analysis could limit the applicability of our findings in contemporary clinical practice. However, our multivariable Cox regression models were also adjusted for mpMRI performance at AS begin, as recommended by EAU guidelines [1]. In consequence, our results are not a product of different AS protocols used for PNBs vs. biopsy naïve patients. Indeed, during follow-up, no meaningful differences in number of repeated biopsies or repeated mpMRI scans were observed between PNBs vs. biopsy naïve patients. Third, we were unable to stratify PNBs patients according to the type of prostate biopsies performed before PCa diagnosis. Specifically, information about PNBs schemes, techniques (systematic vs. targeted cognitive vs. targeted fusion vs. RM-guided) or approaches (transrectal vs. transperineal) are missing. However, the median (IQR) number of cores performed during PNBs was 13 (12–15) and, in consequence, prostate undersampling is unlikely. Fourth, we used specific AS inclusion criteria. In consequence, our results could not be applicable in other AS protocols. Therefore, external validation of our findings is needed for implementing the systematic consideration of PNBs status in AS protocols.

## Conclusion

PNBs status is associated with lower rates of ISUP GG upgrading during AS for PCa. The number of PNBs and time from last PNB to PCa diagnosis (≤ 18 months) appear also to be critical for patient selection.

## Supplementary Information

Below is the link to the electronic supplementary material.

Supplementary Fig. 1 Kaplan–Meier plots with Log-rank test depicting any-cause and ISUP GG upgrading survival according to time from last PNB to AS begin: a any-cause survival in biopsy naïve vs. last PNB > 18 months vs. last PNB ≤ 18 months patients; b ISUP GG upgrading survival in biopsy naïve vs. last PNB > 18 months vs. last PNB ≤ 18 months patients. AS: active surveillance; PNBs: previous negative biopsies; ISUP GG: International Society of Urological Pathology grade group. (PDF 62 KB)

Supplementary file2 (DOCX 24 KB)

Supplementary file3 (DOCX 23 KB)

## References

[1] Mottet N, Bastian P, Bellmunt J et al (2020) Eau-Eanm-Estro-Esur-Siog: guidelines on prostate cancer. In: European Association of Urology. Eur Assoc Urol Guidelines Office, Arnhem, The Netherlands, pp 1–182

[2] Bokhorst LP, Valdagni R, Rannikko A (2016). A decade of active surveillance in the prias study: an update and evaluation of the criteria used to recommend a switch to active treatment. Eur Urol.

[3] Klotz L, Vesprini D, Sethukavalan P (2015). Long-term follow-up of a large active surveillance cohort of patients with prostate cancer. J Clin Oncol.

[4] Moschini M, Carroll PR, Eggener SE (2017). Low-risk prostate cancer: identification, management, and outcomes. Eur Urol.

[5] Tosoian JJ, Trock BJ, Landis P (2011). Active surveillance program for prostate cancer: an update of the Johns Hopkins experience. J Clin Oncol.

[6] Lee MC, Dong F, Stephenson AJ (2010). The epstein criteria predict for organ-confined but not insignificant disease and a high likelihood of cure at radical prostatectomy. Eur Urol.

[7] Adamy A, Yee DS, Matsushita K (2011). Role of prostate specific antigen and immediate confirmatory biopsy in predicting progression during active surveillance for low risk prostate cancer. J Urol.

[8] Kearns JT, Faino AV, Newcomb LF (2018). Role of surveillance biopsy with no cancer as a prognostic marker for reclassification: results from the canary prostate active surveillance study[formula presented]. Eur Urol.

[9] Cary KC, Cowan JE, Sanford M (2014). Predictors of pathologic progression on biopsy among men on active surveillance for localized prostate cancer: the value of the pattern of surveillance biopsies. Eur Urol.

[10] Wong LM, Alibhai SMH, Trottier G (2014). A negative confirmatory biopsy among men on active surveillance for prostate cancer does not protect them from histologic grade progression. Eur Urol.

[11] Al Otaibi M, Ross P, Fahmy N (2008). Role of repeated biopsy of the prostate in predicting disease progression in patients with prostate cancer on active surveillance. Cancer.

[12] Bryant RJ, Yang B, Philippou Y (2018). Does the introduction of prostate multiparametric magnetic resonance imaging into the active surveillance protocol for localized prostate cancer improve patient re-classification?. BJU Int.

[13] Luzzago S, de Cobelli O, Mistretta FA (2020). MRI-targeted or systematic random biopsies for prostate cancer diagnosis in biopsy naïve patients: follow-up of a PRECISION trial-like retrospective cohort. Prostate Cancer Prostatic Dis.

[14] Bloom JB, Hale GR, Gold SA (2019). Predicting gleason group progression for men on prostate cancer active surveillance: role of a negative confirmatory magnetic resonance imaging-ultrasound fusion biopsy. J Urol.

[15] Djavan B, Fong YK, Ravery V (2005). Are repeat biopsies required in men with PSA levels ≤4 ng/ml? a multiinstitutional prospective European study. Eur Urol.

[16] Rosenbaum CM, Mandel P, Tennstedt P (2017). The impact of repeat prostate biopsies on oncologic, pathological and perioperative outcomes after radical prostatectomy. J Urol.

[17] Djavan B, Ravery V, Zlotta A (2001). Prospective evaluation of prostate cancer detected on biopsies 1, 2, 3 and 4: when should we stop?. J Urol.

[18] Elshafei A, Nyame Y, Kara O (2016). More favorable pathological outcomes in men with low risk prostate cancer diagnosed on repeat versus initial transrectal ultrasound guided prostate biopsy. J Urol.

[19] Kopp RP, Stroup SP, Schroeck FR (2012). Are repeat prostate biopsies safe? A cohort analysis from the SEARCH database. J Urol.

[20] Luzzago S, Musi G, Catellani M (2018). Multiparametric magnetic-resonance to confirm eligibility to an active surveillance program for low-risk prostate cancer: intermediate time results of a third referral high volume centre active surveillance protocol. Urol Int.

[21] Luzzago S, Catellani M, Di Trapani E (2020). Confirmatory multiparametric magnetic resonance imaging at recruitment confers prolonged stay in active surveillance and decreases the rate of upgrading at follow-up. Prostate Cancer Prostatic Dis.

[22] Barentsz JO, Richenberg J, Clements R (2012). ESUR prostate MR guidelines 2012. Eur Radiol.

[23] Turkbey B, Rosenkrantz AB, Haider MA (2019). Prostate imaging reporting and data system version 2.1: 2019 update of prostate imaging reporting and data system version 2. Eur Urol.

[24] Weinreb JC, Barentsz JO, Choyke PL (2016). PI-RADS Prostate imaging–reporting and data system: 2015, version 2. Eur Urol.

[25] Nicolosi P, Ledet E, Yang S (2019). Prevalence of germline variants in prostate cancer and implications for current genetic testing guidelines. JAMA Oncol.

[26] Giri VN, Knudsen KE, Kelly WK (2020). Implementation of germline testing for prostate cancer: philadelphia prostate cancer consensus conference 2019. J Clin Oncol.

[27] Carter HB, Helfand B, Mamawala M (2019). Germline mutations in atm and brca1/2 are associated with grade reclassification in men on active surveillance for prostate cancer(figure presented.). Eur Urol.

[28] Lamy PJ, Allory Y, Gauchez AS (2018). Prognostic biomarkers used for localised prostate cancer management: a systematic review. Eur Urol Focus.

[29] Nakanishi H, Groskopf J, Fritsche HA (2008). PCA3 molecular urine assay correlates with prostate cancer tumor volume: implication in selecting candidates for active surveillance. J Urol.

