# Supplementary figures and images for: Association between previous negative biopsies and lower rates of progression during active surveillance for prostate cancer
Source: World J Urol. 2022 Mar 26;40(6):1447–54. doi: 10.1007/s00345-022-03983-8 (PMC9166841; doi:10.1007/s00345-022-03983-8)

A

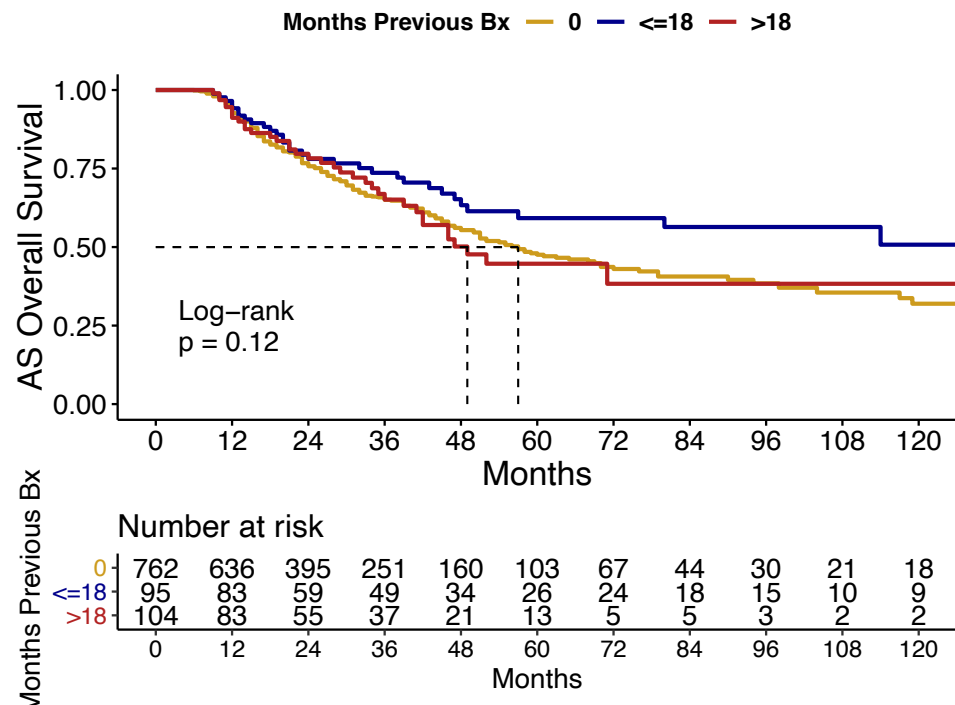

B

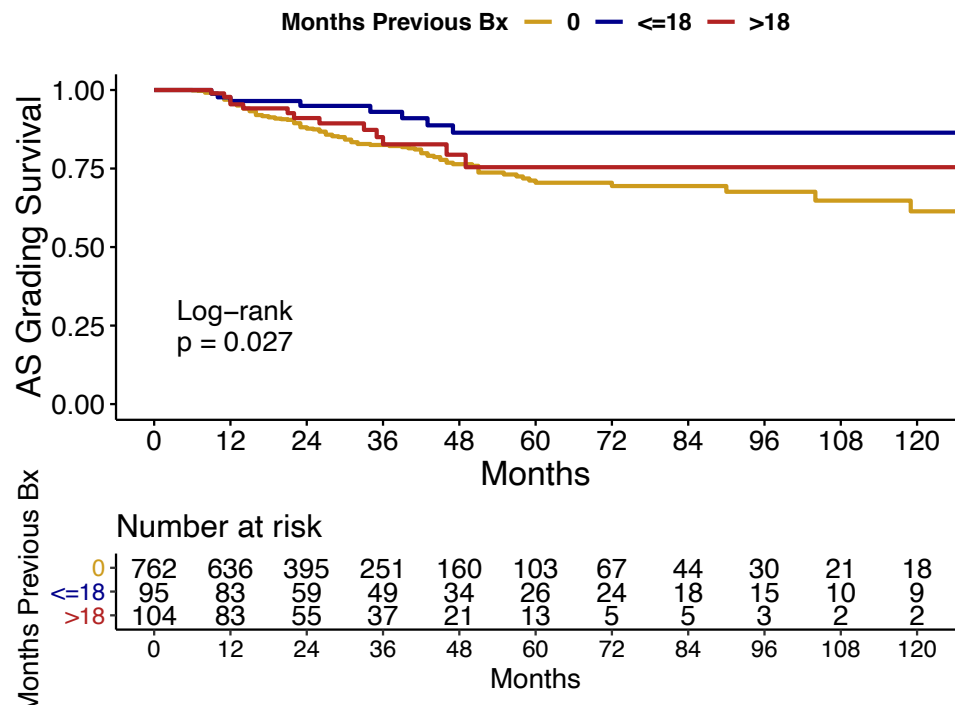

Supplement: Supplementary file 1 — Supplementary Fig. 1 Kaplan–Meier plots with Log-rank test depicting any-cause and ISUP GG upgrading survival according to time from last PNB to AS begin: a any-cause survival in biopsy naïve vs. last PNB > 18 months vs. last PNB ≤ 18 months patients; b ISUP GG upgrading survival in biopsy naïve vs. last PNB > 18 months vs. last PNB ≤ 18 months patients. AS: active surveillance; PNBs: previous negative biopsies; ISUP GG: International Society of Urological Pathology grade group. (PDF 62 KB) [file 345_2022_3983_MOESM1_ESM.pdf]
