# Supplementary material for: Association between previous negative biopsies and lower rates of progression during active surveillance for prostate cancer
Source: World J Urol. 2022 Mar 26;40(6):1447–54. doi: 10.1007/s00345-022-03983-8 (PMC9166841; doi:10.1007/s00345-022-03983-8)
Supplement: Supplementary file 2 — Supplementary file2 (DOCX 24 KB) [file 345_2022_3983_MOESM2_ESM.docx]

**Supplementary Table 1.** Findings at follow-up of 961 patients enrolled in AS between 2008 and 2020. Stratiﬁcation is made according to PNBs status (biopsy naïve vs. PNBs). Data are shown as medians for continuous variables or as counts and percentages (%) for categorical variables.

|  | Overall  (n = 961) | Biopsy naïve  (n = 760; 79.1) | PNBs  (n = 201; 20.9) | p value |
| --- | --- | --- | --- | --- |
| Surveillance biopsies  0  1  2  ≥3 | 341 (35.5)  401 (41.7)  147 (15.3)  72 (7.5) | 259 (34.1)  335 (44.1)  114 (15)  52 (6.8) | 82 (40.8)  66 (32.8)  33 (16.4)  20 (10) | **0.03** |
| Repeat mpMRI scans  0  1  2  ≥3 | 173 (18)  377 (39.2)  230 (23.9)  181 (18.8) | 134 (17.6)  299 (39.3)  190 (25)  137 (18) | 39 (19.4)  78 (38.8)  40 (19.9)  44 (21.9) | 0.3 |
| AS discontinuation causes  No discontinuation  ISUP GG upgrading  Volume upstaging  Rising PSA  Suspicious EPE at mpMRI  Patient preference | 630 (65.6)  142 (14.8)  47 (4.9)  12 (1.2)  70 (7.3)  60 (6.2) | 498 (65.5)  120 (15.8)  42 (5.5)  8 (1.1)  55 (7.2)  37 (4.9) | 132 (65.7)  22 (10.9)  5 (2.5)  4 (2)  15 (7.5)  23 (11.4) | **0.003** |
| Any-cause discontinuation  No  Yes | 630 (65.6)  331 (34.4) | 498 (65.5)  262 (34.5) | 132 (65.7)  69 (34.3) | 0.9 |
| ISUP GG upgrading discontinuation  No  Yes | 819 (85.2)  142 (14.8) | 640 (84.2)  120 (15.8) | 179 (89.1)  22 (10.9) | 0.1 |

Bold values indicate statistical signiﬁcance p<0.05.

PNBs: previous negative biopsies; AS: active surveillance; IQR: interquartile range; mpMRI: multiparametric magnetic resonance imaging; AS: active surveillance; ISUP GG: International Society of Urological Pathology grade group; PSA: prostate speciﬁc antigen; EPE: extra-prostatic extension.
