# Supplementary material for: Association between previous negative biopsies and lower rates of progression during active surveillance for prostate cancer
Source: World J Urol. 2022 Mar 26;40(6):1447–54. doi: 10.1007/s00345-022-03983-8 (PMC9166841; doi:10.1007/s00345-022-03983-8)
Supplement: Supplementary file 3 — Supplementary file3 (DOCX 23 KB) [file 345_2022_3983_MOESM3_ESM.docx]

**Supplementary Table 2

a** Multivariable Cox-regression models predicting any-cause AS discontinuation rates, according to time from last PNB to AS begin (biopsy naïve vs. last PNB ≤18 months vs. last PNB >18 months). All models are adjusted for clinical characteristics and biopsy ﬁndings at patient enrolment: age (years), PSA (ng/ml), cT (cT1c vs. cT2a), confirmatory mpMRI (no vs. yes), number of positive cores at biopsy (1 vs. 2 vs. 3) and ISUP GG at biopsy (1 vs. 2).

| Any-cause discontinuation rates | | |
| --- | --- | --- |
|  | Hazard ratio (HR) [95% CI] | p value |
| Time from last PNB  Biopsy naïve  ≤18 months  >18 months | Ref.  0.7 (0.5-1.05)  1.1 (0.7-1.5) | 0.09  0.8 |

**a** Multivariable Cox-regression models predicting ISUP GG upgrading rates, according to time from last PNB to AS begin (biopsy naïve vs. last PNB ≤18 months vs. last PNB >18 months). All models are adjusted for clinical characteristics and biopsy ﬁndings at patient enrolment: age (years), PSA (ng/ml), cT (cT1c vs. cT2a), confirmatory mpMRI (no vs. yes), number of positive cores at biopsy (1 vs. 2 vs. 3) and ISUP GG at biopsy (1 vs. 2).

| ISUP GG upgrading rates | | |
| --- | --- | --- |
|  | Hazard ratio (HR) [95% CI] | p value |
| Time from last PNB  Biopsy naïve  ≤18 months  >18 months | Ref.  0.4 (0.2-0.9)  0.8 (0.4-1.5) | **0.02**  0.5 |

Bold values indicate statistical signiﬁcance p<0.05.

AS: active surveillance; PNBs: previous negative biopsies; PSA: prostate speciﬁc antigen; cT: clinical T stage; mpMRI: multiparametric magnetic resonance imaging; ISUP GG: International Society of Urological Pathology grade group; CI: confidence interval.
